# Neonatal Sludge: A finding of congenital hypothyroidism

**DOI:** 10.4274/jcrpe.v1i4.54

**Published:** 2010-12-08

**Authors:** Selim Kurtoğlu, Dilek Çoban, Mustafa Ali Akın, Leyla Akın, Ali Yıkılmaz

**Affiliations:** 1 Erciyes University, Department of Neonatology, Kayseri; 2 Erciyes University, Department of Pediatric Endocrinology, Kayseri; 3 Erciyes University, Department of Radiology, Kayseri; +90-352-437 49 01+90-352-437 49 31cobandilek@hotmail.comErciyes University Pediatric Endocrinology, Kayseri, Turkey

**Keywords:** Neonate, sludge, hypothyroidism

## Abstract

Congenital hypothyroidism is one of the most urgent diseases of the neonate. When diagnosed and treated at an early stage, its most important complication, mental retardation, is preventable. The signs of congenital hypothyroidism are nonspecific in neonates. Only 5% of the cases have characteristic clinical findings. One of the most important and earliest signs is prolonged jaundice during the neonatal period. We report herein a case of congenital hypothyroidism, who presented with icterus accompanied with sludge formation into the gallbladder, which disappeared after treatment with L-thyroxine.

**Conflict of interest:**None declared.

## INTRODUCTION

Congenital hypothyroidism is one of the most important diseases of the newborn, which may lead to mental and physical retardation when treatment is delayed or an appropriate dosage of thyroxine is not used. The physical findings of hypothyroidism are nonspecific, and typical clinical features are observed in only 5% of the neonatal cases.^1^ The most alarming and earliest sign is jaundice, especially when it is prolonged, during the neonatal period.^2^ Unconjugated or conjugated hyperbilirubinemia and prolonged physiologic icterus may be observed in infants with congenital hypothyroidism.^3, 4, 5, 6, 7^ We report herein, a case of congenital hypothyroidism who presented with icterus accompanied with sludge formation in the gallbladder.

## CASE REPORTS

A 3 day-old male infant was admitted to our hospital with jaundice which started on the second day of his life. He was born by cesarean section at 36 weeks of gestation. His mother was healthy and 24 yearsold. During her pregnancy she had used non-iodinized salt. On admission the infant’s weight was 2800 g, length 48 cm, head circumference 36 cm. His skin and sclera were icteric. Anterior fontanel was 4x5 cm and posterior fontanel 1x1 cm; both measurements were larger than the expected dimentions for that age. The patient had abdominal distention and hepatomegaly, palpable 2 cm below costal margin. Laboratory findings were as follows: Hemoglobin: 13.2 g/dl, Htc: 41.4%, reticulocyte count: 3%, total bilirubin: 27.2 mg/dl, conjugated bilirubin: 3.9 mg/dl, AST: 74 IU/L (0-40), ALT: 43 IU/L (0-40), GGT: 240 IU/L (0-60), LDH: 2153 IU/L (100-190). There was no hemolysis on the blood smear and no blood group and subgroup incompatibility was detected. Other laboratory studies did not reveal any abnormalities. 

**Figure 1a:**
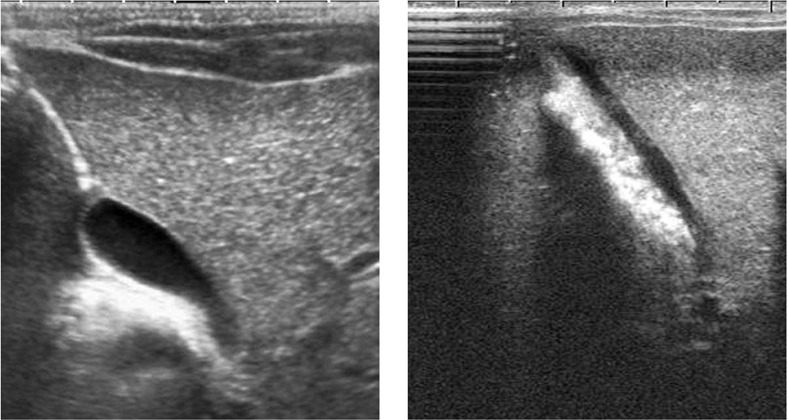
Longitudinal sonogram of the gall bladder shows hyperechoic biliary sludge filling more than half of the gall bladder with posterior acoustic shadow (a). Another sonogram, obtained after treatment, shows complete resolution of the biliary sludge (b).

The patient underwent intensive phototherapy. After four hours, the total and conjugated bilirubin levels were reduced to 20.9 and 3.1 mg/dl, respectively. As the infant had conjugated bilirubinemia, investigations to exclude TORCH infections, metabolic disorders, alfa-1-antitripsin deficiency, anatomic anomalies and thyroid dysfunction were carried out as part of the differential diagnostic work-up. No pathology was detected, except for thyroid function tests. Results of the thyroid function tests suggested primary hypothyroidism [free T4: 9.65 pg/ml (9-26 pg/ml), TSH: 49.90 mIU/ml (0.9-7.7 mU/ml), thyroglobulin: 698.23 ng/ml (12-113 ng/ml)]. Thyroid volume was 1.78 ml (normal median value 0.79 ml^8^). Urinary iodine level was 30 μg/L (normal value above 100 μg/L^8^). The hepatobiliary ultrasonography showed sludge in the gallbladder (Figure 1a). Sodium Lthyroxine (10 μg/kg/day) and lugol (100 μg/day) were administered. After treatment, thyroid volume was reduced to 0.99 ml within seven days and sludge disappeared within fourteen days (Figure 1b). At that time thyroid hormone levels and direct/indirect bilirubin levels were as follows: free T4: 31.84 pg/ml (9-26 pg/ml), TSH: 0.37 mIU/ml (0.9-7.7 mU/ml), thyroglobulin: 208.09 ng/ml (12-113 ng/ml), total bilirubin: 4.4 mg/dl, conjugated bilirubin: 0.9 mg/dl, AST: 28 IU/L (0-40), ALT: 26 IU/L (0-40). After 4 months of treatment, thyroid hormone levels were as follows: free T4: 15.41 pg/ml, TSH 1.13 μIU/ml and urinary iodine: 130 μg/L. At present, the patient’s follow-up at the pediatric endocrinology department is ongoing.

## DISCUSSION

Congenital hypothyroidism, and primary hypothyroidism in particular, is a worldwide problem. Although thyroid dysgenesis is the most common cause of primary hypothyroidism, iodine deficiency is still prominent in many parts of the world. Turkey, including the Kayseri region, has been recognized as an area where moderate to severe iodine deficiency is prevalent.^8^ In the present case, we considered iodine deficiency as a result of low urinary iodine excretion, large thyroid volume (>1.5 ml^9^) and high serum thyroglobulin and TSH concentrations.

Early onset conjugated and prolonged physiologic hyperbilirubinemia can be observed in newborns with hypothyroidism. ^3, 4, 5, 6, 7^ Neonatal cholestatic hepatitis is often related to congenital combined pituitary hormone deficiency.^10^ Isolated cortisol deficiency^11^ and pseudohypoaldosteronism12 are among other possible causes of cholestatic hepatitis. Sludge formation may also be associated with sepsis, parenteral nutrition, severe hemolytic diseases, metabolic disease, some drugs and pyloric stenosis.^13, 14, 15, 16, 17^ We confirmed hypothyroidism in our patient after excluding other causes of sludge formation. Sludge disappeared after L-thyroxine treatment. At follow-up, there was no decrease in the patient’s hemoglobin levels and bilirubin levels were not elevated.

The mechanism of sludge formation in hypothyroidism has not been fully explained but some reports have shown that thyroxine and triiodothyronine have an inhibitory effect on Oddi sphincter’s contractility.^18, 19, 20^ off Oddi regulates bile flow. Thyroxine and triiodothyronine have a prorelaxing effect on sphincter of Oddi.^20^ The absence of the prorelaxing effect of thyroxine results in delayed emptying of the biliary tract.^18, 19, 20^ In addition, thyroid hormones also affect cholesterol metabolism. Biliary secretion of cholesterol is reduced in hypothyroidism; bile may also become supersatured with cholesterol causing sludge or gallstone formation.^19^ There have been some reports of common bile duct stones associated with hypothyroidism.^18, 19, 20, 21^

It is important to underline that sludge formation may occur in neonatal hypothyroidism and should be evaluated in routine controls. However, further observations in support of this view are warranted. To our knowledge, there are no previous reports of sludge formation and jaundice associated with neonatal hypothyroidism.
